# A Case of a Rare, Incidental Discovery of Fibrous Obliteration of an Appendiceal Diverticulum

**DOI:** 10.7759/cureus.26681

**Published:** 2022-07-09

**Authors:** Joshua K Jenkins, Colton A Morrow, Shweta Chaudhary, Jeffery H Brashear

**Affiliations:** 1 Research, Lincoln Memorial University-DeBusk College of Osteopathic Medicine, Harrogate, USA; 2 Pathology, Hazard Appalachian Regional Healthcare (ARH) Regional Medical Center, Hazard, USA; 3 General Surgery, Hazard Appalachian Regional Healthcare (ARH) Regional Medical Center, Hazard, USA

**Keywords:** vermiform appendix, fibrous obliteration, appendiceal diverticulosis, diverticulosis, appendix

## Abstract

Diverticulosis of the appendix (DA) is rare and frequently found incidentally. Some cases are discovered after presenting with similar symptomatology to acute appendicitis, whereas other cases may be completely silent. Fibrous obliteration (FO) is a histologic finding indicative of cellular proliferation secondary to relapses of subclinical inflammatory processes. We report a case of a 75-year-old female with a history of chronic, intermittent abdominal pains who presented to the general surgery clinic after an abnormal thickening of the appendix was discovered on abdominal and pelvic computed tomography imaging. The patient underwent laparoscopic appendectomy for suspicion of malignancy. The histologic evaluation of the specimen demonstrated a diverticulum at the distal end of the appendix with FO of the lumen. We suspect the chronic nature of her disease course may have led to the FO of the diverticulum. An extensive literature search was performed, which revealed no other cases of FO of appendiceal diverticula. This may be the first case of diverticulosis of the appendix with FO in the English medical literature. If DA is discovered early with non-invasive imaging, surgical excision should be performed prophylactically as an association with an increased risk of perforation and neoplastic progression has been found.

## Introduction

Diverticulosis of the appendix (DA), or appendiceal diverticular disease (ADD), is a rare occurrence with an incidence ranging from 0.004% to 2.1% [1-5]. Since it was first characterized in 1893, this condition has been characterized as either congenital or acquired [2]. The congenital form is extremely rare and consists of herniation of all layers of the appendiceal wall, constituting a true diverticulum. The acquired form is more prevalent and consists only of mucosal herniations through a muscular defect on the mesenteric border of the appendix, constituting a false diverticulum [6].

DA can be completely asymptomatic or present similarly to acute appendicitis if the diverticulum becomes inflamed. Most cases of DA are discovered incidentally during post-operative histopathologic evaluation of the specimen or during intra-operative laparoscopy [4,5,7]. Diagnosis can also be attained through barium enema if there is suspicion of DA in the clinical setting [4]. The current evidence is limited to only case reports and retrospective studies; however, the current recommendation is to pursue appendectomy as DA increases the risk for future appendiceal perforation and coexisting appendiceal neoplasms [8,9-11].

Fibrous obliteration (FO) is a proliferative lesion that was first described by Masson in 1928 [12,13]. He examined the specimens under electron microscopy, which discovered the cells to contain secretory granules of serotonin and somatostatin in their cytoplasm. The pathogenesis of this phenomenon is unknown; however, various studies hypothesize the etiology as secondary to recurrent lapses of inflammation. This process causes hyperplasia of the neuroendocrine cells in the lamina propria and submucosa of the appendix wall. Repetition of this subclinical inflammatory process yields fibrosis as an end-product. Many cases of FO of the appendix can also mimic appendicitis-like symptoms [12,13].

Here, we report a case of an elderly woman who was incidentally found to have FO of an appendiceal diverticulum after laparoscopic appendectomy for a suspected mucocele neoplasm of the appendix. The patient was found to have a thickened appendix on abdominal and pelvic computed tomography (CT) imaging after several years of non-specific complaints of abdominal pain.

## Case presentation

**Figure 1:**
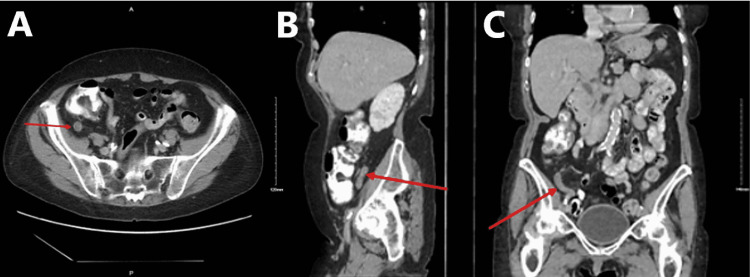
Computed tomography image of the abdomen and pelvis with the oral contrast demonstrating a thickened appendix of approximately 11 mm in the (A) transverse plane, (B) sagittal plane, and (C) coronal plane

A 75-year-old Caucasian female presented to her primary care provider’s (PCP’s) office with complaints of intermittent, mild episodes of non-specific abdominal pains for approximately five years. She also noted occasional post-prandial nausea with the consumption of solid and liquid food products and globus sensation, which she attributed to a history of sliding hiatal hernia. Her past medical history was also significant for gastroesophageal reflux disease, chronic obstructive pulmonary disease, and hypertension. Her surgical history included hysterectomy, cholecystectomy, and esophagogastroduodenoscopy (EGD), which revealed the sliding hiatal hernia four years ago. Her PCP ordered abdominal and pelvic CT imaging, which was significant for a thickened appendix with an approximated diameter of 11 mm (Figures 1A-1C). She was referred to the services of general surgery for further evaluation.

**Figure 2:**
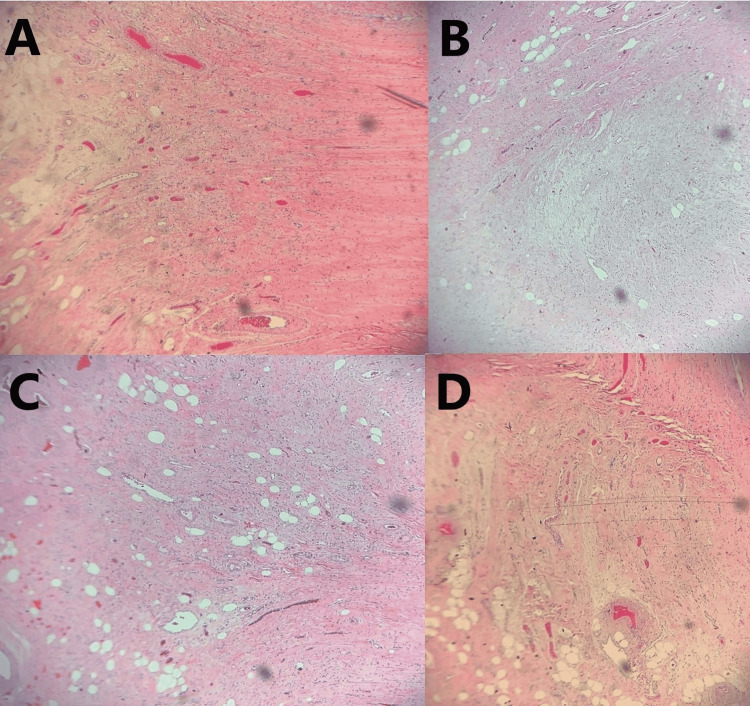
(A-D) Hematoxylin and eosin (H&E) sections demonstrating fibrous obliteration of a diverticulum arising from the tip of the appendix with the invasion of the muscularis propria

**Figure 3:**
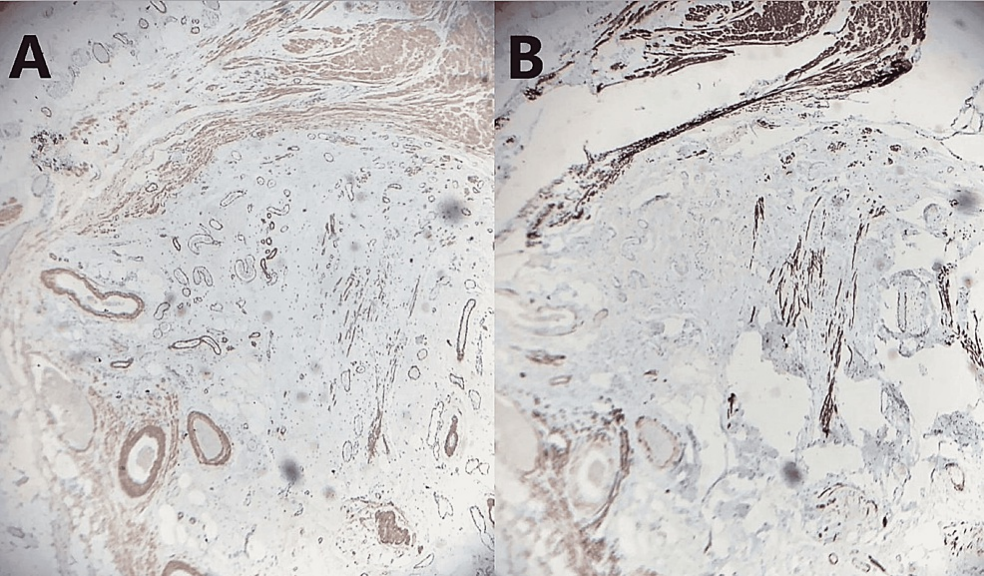
(A) IHC stain demonstrating diverticular fibroblasts and blood vessel walls positive for smooth muscle actin (x20); (B) IHC stain of the obliterated appendiceal diverticulum demonstrating negativity for desmin to rule out smooth muscle neoplasms (x20) IHC, immunohistochemical

On arrival at the general surgery clinic, the patient only noted symptoms relating to her hiatal hernia, such as occasional post-prandial nausea and vomiting, but denied current abdominal pain. She also denied fever, chills, loss of appetite, diarrhea, rectal bleeding, and melena. On physical examination, her abdomen was soft, non-distended, and non-tender. McBurney’s point and Rovsing’s sign were both negative. After reviewing the CT imaging, malignant processes, such as mucocele, could not be excluded, and laparoscopic appendectomy was recommended. After discussing options of observation versus surgical intervention, and the risks, benefits, and potential outcomes of each, she consented to the procedure. During the operation, the appendix was found to be grossly enlarged and pale in appearance. The gross specimen was measured at 68 mm in length and 13 mm in diameter. The serosal surface was pink-to-tan in color. Transverse sectioning revealed a grossly edematous mucosa that occluded the lumen. The distal tip of the appendix appeared expanded, giving a gross appearance most consistent with that of a diverticulum. Histologic evaluation of the specimen confirmed diverticulosis of the distal appendix with FO of the diverticulum (Figures 2A-2D). The specimen was sent for specialty oncology evaluation to rule out neoplastic processes. The diagnosis was confirmed as diverticulosis of the distal appendix without evidence of acute appendicitis, mucocele, adenocarcinoma, or neuroendocrine carcinoma (Figures 3A-3B).

## Discussion

DA is a rare occurrence and most frequently occurs as a pseudodiverticula derived from a weakening of the muscularis propria of the appendiceal wall [4]. In contrast, FO of the appendix is much more common than DA. Various studies hypothesize the etiology of FO as being secondary to recurrent lapses of inflammation. Repetition of this subclinical inflammatory process yields fibrosis as an end-product [12,13]. We suspect that the chronic, five-year course of the patient's intermittent, mild episodes of non-specific abdominal pains may have been derived from a mild inflammatory process of the appendiceal diverticulum, which may have led to the FO observed in the specimen. To our knowledge, the present case is the only case of FO of an appendiceal diverticulum in the English medical literature.

**Figure 4:**
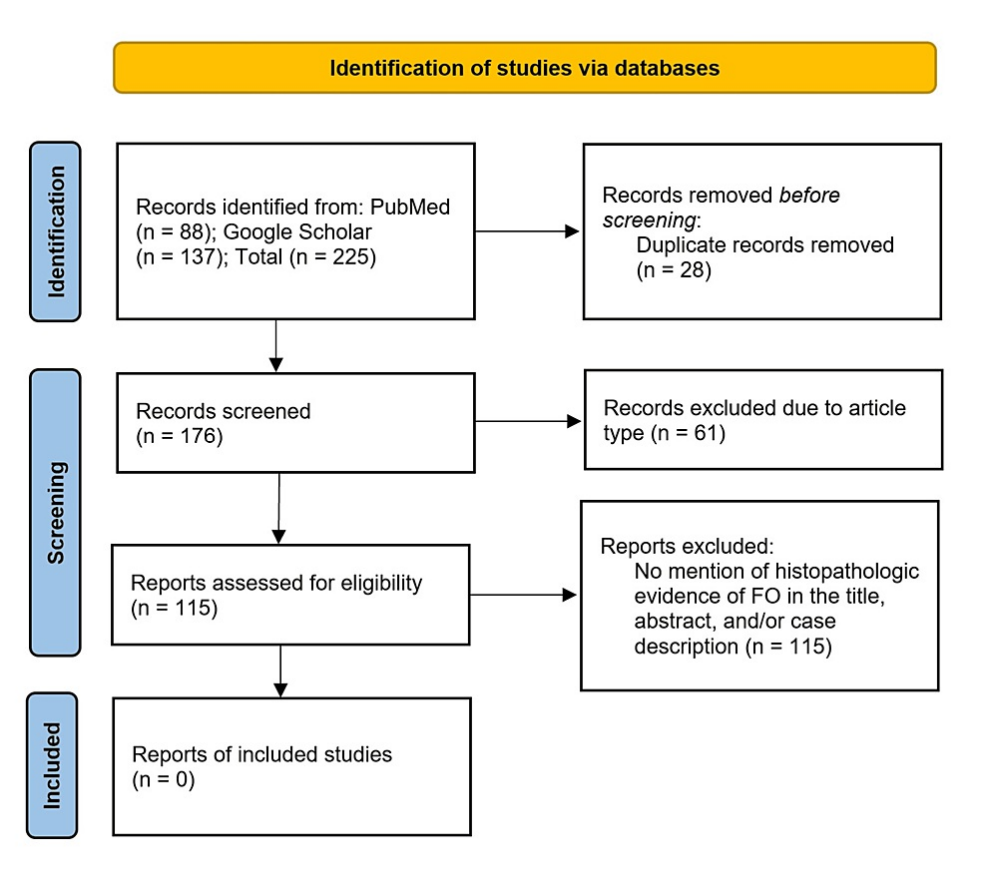
PRISMA flow diagram demonstrating the systematic approach to the literature review PRISMA, Preferred Reporting Items for Systematic Reviews and Meta-Analyses; FO, fibrous obliteration

Our literature search was performed using the Preferred Reporting Items for Systematic Reviews and Meta-Analyses (PRISMA) 2020 guidelines with the assistance of Harzing's Publish or Perish® software (free, open-source) [14]. The search terms included ("appendix") AND ("diverticula" OR "diverticuli" OR "diverticulosis") in the PubMed (n = 88) and Google Scholar (n = 137) databases. In total, 28 of these cases were duplicates and were removed prior to screening. The 176 remaining articles were filtered by article type to include case reports, retrospective analyses, and journal articles. A total of 61 cases were excluded due to article type (i.e., citations, letters). The remaining 115 appendiceal diverticulum cases were then screened for the mention of histopathologic evidence of FO in the title, abstract, and case description. No cases of FO of an appendiceal diverticulum were found in this search (Figure 4).

Kalyneck described the first case of DA in 1893 [2]. Since then, it has been characterized as either congenital or acquired. To differentiate these categories, congenital diverticula are usually located on the anti-mesenteric edge and involve all layers of the appendiceal wall (i.e., mucosa, submucosa, muscularis, and serosa). Acquired diverticula are small, ranging from 2 to 5 mm in size, and are typically located in the distal third of the appendix on the mesenteric edge [15-16]. In the present case, the expanded distal end of the appendix and histologic evidence were most consistent with an acquired diverticulum.

A few studies have demonstrated an association between DA and neoplastic processes. Lim et al. performed a systematic review and meta-analysis, which revealed the prevalence of neoplasia in appendix specimens with DA to be 26.94%, compared to a 1.28% prevalence of neoplasia in specimens without DA [9]. This study added to a retrospective study by Kallenbach et al., who found that 43.9% (17/39) of appendix specimens with DA coexisted with neoplastic processes. In this study, DA was found to be associated with low-grade mucinous neoplasia (n = 2), sessile serrated adenoma/polyp (n = 2), lymphoid hyperplasia (n = 1), fibrosis of the periappendicular region (n = 2), adenocarcinoma of the appendix (n = 1), and colorectal carcinoma (n = 4) [10]. In the present case, there was initial concern for mucocele as a potential malignant process given the clinical scenario. Histologic examination, however, confirmed otherwise. The specimen was also sent out for specialist oncologic evaluation, which confirmed no evidence of mucocele, adenocarcinoma, or neuroendocrine carcinoma.

## Conclusions

In conclusion, DA is a rare entity often discovered incidentally with an asymptomatic presentation or with symptomatology similar to acute appendicitis. Here, we presented the case of FO of a diverticulum of the appendix in a woman who had been experiencing intermittent episodes of subclinical abdominal pain for five years. We suspect the chronic nature of her disease course may have led to the FO of the diverticulum. This may be the first case of DA with FO in the English medical literature. Diagnosis of this entity prior to operative intervention is challenging as it may be completely silent. If DA is found early through the use of barium enema or CT imaging, surgical excision should be performed prophylactically as an association with an increased risk of perforation and neoplastic progression has been found previously.
